# Behavioral health as a palliative care priority in long-term services and supports: A cross-sectional study of staff

**DOI:** 10.1017/S1478951525100977

**Published:** 2025-11-04

**Authors:** Molly A. Nowels, Rose L. Carlson, Manali Saraiya, Catherine A. Riffin, Evan Plys, M. Carrington Reid, Taimur Mirza, Ronald D. Adelman, Mark Aaron Unruh, Daniel Shalev

**Affiliations:** 1 Division of Geriatrics and Palliative Medicine, Department of Medicine, Weill Cornell Medicine, New York, NY, USA; 2 Department of Psychiatry, Harvard Medical School, Boston, MA, USA; 3 ArchCare, New York, NY, USA; 4 Department of Population Health Sciences, Weill Cornell Medicine, New York, NY, USA

**Keywords:** Long-term care, long-term services and supports, behavioral health, nursing homes, palliative care

## Abstract

**Objectives.:**

Behavioral health needs are highly prevalent among individuals receiving long-term services and supports (LTSS), yet palliative care (PC) models in these settings often underemphasize psychiatric symptom management. This study explores interdisciplinary staff perspectives on behavioral health as a core domain of PC across nursing home and Program of All-Inclusive Care for the Elderly (PACE) sites.

**Methods.:**

We conducted a secondary analysis of a multi-site survey assessing PC needs across 13 LTSS sites within a large health system in New York State. We examined 5 survey items related to psychiatric symptom management, analyzing frequency, comfort, perceived benefit, and training interest. Multivariable logistic regression was used to assess associations between staff characteristics and behavioral health-related outcomes.

**Results.:**

Among 597 respondents, 60.5% reported that over half of their patients could benefit from psychiatric symptom management, and nearly half (49.2%) reported managing such symptoms weekly or more. Forty percent identified psychiatric symptom management as one of the top three ways PC specialists could help their patients, and 44.6% expressed interest in further behavioral health training as part of further PC training. Prior professional experience with PC was associated with greater recognition of behavioral health needs among patients (aOR 1.6), greater likelihood of managing psychiatric symptoms (aOR 2.0), and greater comfort doing so (aOR 1.5).

**Significance of results.:**

Behavioral health emerged as a salient and frequently encountered domain of serious illness care among LTSS staff, particularly in nursing home and PACE settings. Staff with prior PC experience were more engaged and confident in addressing psychiatric symptoms. Findings underscore the need for PC models in LTSS to better integrate behavioral health – through training, interdisciplinary collaboration, and care delivery redesign – to meet the complex needs of medically and psychiatrically vulnerable populations.

## Introduction

Palliative care (PC) is a specialized interdisciplinary care model that aims to improve the quality of life for individuals with serious illness and their caregivers by addressing physical, emotional, social, and spiritual needs (Ferrell et al. 2018). Behavioral health, including psychiatric symptom management, constitutes a core component of high-quality PC (Ferrell et al. 2018). In practice and research, however, behavioral health symptom management within PC remains underdeveloped relative to physical symptoms (Nowels et al. 2023; Shalev et al. 2023, 2024).

This gap may be especially evident in long-term services and supports (LTSS), which encompass a wide range of care models for medically complex individuals who require assistance with daily activities and ongoing medical management. LTSS includes both institutional care (e.g., nursing homes and community-based programs (e.g., adult day health and Programs of All-Inclusive Care for the Elderly [PACE]). For brevity, we refer to these collectively as long-term care (LTC) throughout. The LTC population is growing rapidly: the 1.3 million U.S. residents currently in nursing homes are expected to double by 2030 (FastStats 2024; Kelley and Morrison 2015). Across settings, LTC recipients experience a high burden of serious illness, often accompanied by poorly managed symptoms and a disproportionately high likelihood of receiving inappropriate end-of-life care (Esteban-Burgos et al. 2021; Hermans et al. 2017; Koroukian et al. 2023; Stephens et al. 2018). Despite this burden, PC has been only modestly integrated into models of LTC (Cole et al. 2024a, 2024b; Haroen et al. 2025; Kelley and Morrison 2015).

One barrier to integration is that PC delivery models designed for ambulatory or acute care do not translate easily to LTC. For example, nursing homes and PACE programs often rely on external contractors to provide specialty PC, which can limit access to timely and consistent care. Integrating PC into LTC will therefore likely require adapting care models to align with LTC workforce composition, delivery systems, and patient needs.

Behavioral health represents a particularly urgent domain for such adaptation. Depression, anxiety, serious mental illnesses such as schizophrenia and bipolar affective disorder, and dementia-related neuropsychiatric symptoms are highly prevalent in LTC residents. About one-third of nursing home residents have a behavioral health diagnosis other than dementia, and nearly half have dementia with accompanying neuropsychiatric symptoms (Fashaw et al. 2020). Furthermore, approximately 1 in 5 nursing home residents has a serious mental illness (e.g., schizophrenia, bipolar affective disorder); a proportion that has increased steadily for the past 2 decades (Fashaw et al. 2020; Hua et al. 2021; Laws et al. 2022; Muralidharan et al. 2019). Similarly, about 60% of PACE enrollees have behavioral health diagnoses (Fleet et al. 2025). Behavioral health conditions frequently co-occur with serious medical illness, amplifying symptom burden, impairing quality of life, complicating care delivery, and contributing to staff strain and systems-level challenges.

To meet the needs of LTC populations, PC models must explicitly integrate behavioral health, both through interdisciplinary team structures and by training staff in primary PC behavioral health symptom management. However, little is known about how LTC staff view psychiatric symptom management as it relates to PC. To address this gap, we conducted a secondary analysis of a multi-site PC needs assessment survey. Our analysis focuses on nursing home and PACE interdisciplinary staff perspectives related to behavioral health as a component of PC, including their attitudes, self-reported practices, comfort levels, and perceived training needs. These findings aim to inform the development of more responsive PC models with behavioral health components tailored to the needs of individuals receiving LTC services.

## Methods

### Survey design, development, and dissemination

We developed a survey to assess staff members’ attitudes, knowledge, and perceived clinical and training needs related to PC across a network of nursing homes and PACE sites in New York State. The 27-item survey had 4 domains: (1) respondent demographics and professional background (10 items); (2) attitudes toward, knowledge of, and prior exposure to PC (6 items); (3) current participation in PC delivery (5 items); and (4) PC-related clinical and training needs (6 items). The instrument (see Supplement 1) was developed using the National Consensus Project Clinical Practice Guidelines for Quality Palliative Care as a conceptual framework (Ferrell et al. 2018). The survey was distributed in May and June of 2023viaemailtoallstaffmembers(*N* = 1,193)at13sites,including 7 nursing homes, 4 PACE sites, and 2 home- or community-based care programs for older adults with nursing home–level needs.

### Data analysis

We analyzed data from 5 survey questions (highlighted in Supplement 1) that addressed respondents’ attitudes and perspectives on behavioral health.

To facilitate statistical analysis, we dichotomized each question as follows: 1) percentage of residents/patients who could benefit from additional psychiatric symptom management (< 50% vs. ≥ 50%), 2) frequency with which psychiatric symptom management is provided to patients at their facility (< vs. ≥ weekly), 3) respondent’s comfort managing psychiatric symptoms (NA, very uncomfortable, uncomfortable, or neither comfortable nor uncomfortable vs. comfortable or very comfortable), 4) whether the provider indicated that psychiatric symptom management was one of the three most common ways a PC specialist could help their patients (yes vs. no), and 5) whether the provider indicated that they want to strengthen/expand their psychiatric symptom management skills (yes vs. no). As a note, we use the term psychiatric rather than behavioral health in the results to match the wording of the survey, in which psychiatric was used because of its more frequent use in clinical settings.

We characterized the sample using frequencies and percentages relative to each independent variable: provider demographics (i.e., race/ethnicity (Black, Asian American/Pacific Islander [AAPI], Hispanic/Latino, White, other/not reported), age (30 or younger, 31–40, 41–50, 51–60, 61 or older, prefer not to answer), and gender (man, woman, non-binary/unknown)), professional discipline (grouped by role similarity as physician/NP/PA, social worker/case manager, RN, CNA/LPN, PT/OT/SLP, and Other), and setting (i.e., LTC, PACE, other, multiple settings). We used multivariable logistic regression to identify demographic and professional correlates of each outcome. Summary statistics and analyses were conducted in R version 4.4.1.

## Results

A total of 597 staff members from across the healthcare system completed the survey for a response rate of 50.0%. More than two-thirds of respondents worked in either the nursing home setting (*n* = 225, 37.7%) or multiple settings (*n* = 204, 34.2%). Most respondents identified as women (*n* = 475, 79.6%) and were older than 40 (*n* = 359, 60.1%). The most frequently reported racial/ethnic group was Black (*n* = 220, 36.9%), followed by White (*n* = 150, 25.1%). The most common professional roles were certified nursing assistants or licensed practical nurses (CNAs/LPNs) (*n* = 198, 33.2%) and registered nurses (RNs) (*n*= 131, 21.9%). Most respondents had practiced for at least 5 years (*n* = 413, 69.2%). See Table 1 for respondent characteristics.

### Patients who could benefit from psychiatric symptom management

A majority (*n* = 361, 60.5%) of respondents indicated that at least 50% of their patients could benefit from psychiatric symptom management. Compared to respondents over 61 years old, younger staff members were more likely to hold this view. Those under 30 had an adjusted odds ratio of 3.51 (95% CI: 1.4–9.1) and those aged 31–40 had an aOR of 2.7 (95% CI: 1.3–6.0). Furthermore, compared to RNs, physicians (MD/DOs), NPs, and PAs were more than 5 times more likely to endorse a need for psychiatric symptom management among more than half of their patients (aOR = 5.4, 95% CI [1.6, 25.3]). Providers with professional experience with PC were 62% more likely to endorse this opinion about their patients compared to those without any experience with PC (aOR = 1.6, 95% CI [1.1, 2.4]). See Table 2 for responses to psychiatric symptom-related items.

### Frequency of managing psychiatric symptoms

Nearly half (*n* = 294, 49.2%) of survey respondents reported that they manage psychiatric symptoms at least weekly. Physicians, NPs, and PAs were more likely than RNs to do so (aOR = 10.9 95%CI[2.1, 201.5]). Likewise, providers with either personal or professional PC experience were more likely than those with no previous PC experience to manage psychiatric symptoms at least weekly (personal experience: aOR = 1.7, 95%CI[1.1, 2.6]; professional experience: aOR = 2.0, 95%CI [1.3., 3.0]).

### Comfort managing psychiatric symptoms

Nearly half (*n* = 291, 48.7%) of respondents reported being comfortable or very comfortable managing patients’ psychiatric symptoms. Professional experience with PC was associated with greater comfort (aOR = 1.5, 95%CI[1.0, 2.4]).

### Palliative care specialists could help with psychiatric symptom management

Forty percent of respondents (*n* = 241) identified psychiatric symptom management as one of the top three ways PC specialists could support their patients. Staff working in PACE settings were more likely to agree that PC specialists could provide psychiatric symptom management (aOR = 2.2, 95% CI [1.1, 4.5]).

### Desire to strengthen and expand psychiatric symptom management skills

Almost half (*n* = 266, 44.6%) of respondents expressed interest in undergoing training in psychiatric symptom management. Black (vs. white) respondents were 84% more likely than their white counterparts to desire additional training in psychiatric symptom management (aOR = 1.8, 95%CI[1.1, 3.0]).

## Discussion

This study examined interdisciplinary staff perspectives on behavioral health within LTC settings and found that behavioral health is perceived as both a common clinical need and a key domain in which PC can contribute. Most staff reported that over half of their patients could benefit from psychiatric symptom management, and nearly half indicated that they manage psychiatric symptoms at least weekly. Furthermore, many respondents identified psychiatric symptom management as an area where PC specialists could support patient care, and nearly half expressed a desire to strengthen their skills in this area.

One of the most striking findings was the association between prior professional exposure to PC and engagement with behavioral health care. Compared to their peers, staff with PC experience were significantly more likely to recognize psychiatric symptoms among their patients, report more frequently managing these symptoms, and feel comfortable in doing so. These findings suggest that exposure to PC may not only equip staff with specific skills but also attune them to behavioral health needs as integral to serious illness care.

Our findings highlight that frontline staff recognize behavioral health as an essential, not optional, component of serious illness care. For PC to meet the needs of LTC populations effectively, models must highlight behavioral health as a core component of this model, both through interdisciplinary collaboration and through workforce training in primary palliative psychiatry skills.

This study has several limitations. It relies on cross-sectional, self-reported data and may be subject to response and social desirability bias. The survey was conducted within a single large health system in New York State, which may limit generalizability to other geographic or organizational contexts. Furthermore, the survey instrument was developed for internal quality improvement and has not been externally validated. While our analysis focused on staff perspectives, we did not capture the views of patients or family caregivers, whose experiences with behavioral health and PC needs are critical to a complete understanding of care quality in these settings.

Our findings have several practical and policy implications. First, efforts to expand PC in LTC settings should prioritize behavioral health. This may include structured collaboration with behavioral health professionals, including psychiatrists, psychologists, and licensed clinical social workers. Second, interdisciplinary team members – especially CNAs, LPNs, and RNs – would likely benefit from targeted training in psychiatric symptom management as a core component of primary PC training. Few PC educational interventions in LTC settings include behavioral health, despite a general call for greater behavioral health training for LTC staff (Lamppu and Pitkala 2021; Molinari et al. 2008; Muralidharan et al. 2019). However, appropriately focused training may enhance the association between engagement with meeting behavioral health needs and prior PC experience, which we found. Even limited exposure to PC appears to be associated with more proactive engagement in psychiatric symptom management, suggesting a powerful opportunity for synergistic effects in primary PC training.

Innovative care models, such as embedding behavioral health specialists, interdisciplinary psychiatric-PC consultation, or telehealth-based collaboration, constitute promising solutions for bridging current gaps (Cheung et al. 2019; Shalev et al. 2020, 2021; Wozniak et al. 2021). Fully integrating behavioral health into PC not only addresses patient needs but also has the potential to shift care culture by equipping and empowering frontline staff to recognize and respond to psychiatric symptoms as part of routine care. Ultimately, future research should assess the implementation and impact of these models on outcomes, including symptom burden, staff confidence, avoidable hospital transfers, and the quality of life for patients and caregivers.

As LTC programs care for growing numbers of individuals with co-occurring medical and psychiatric illness, PC models that fully integrate behavioral health will be essential to providing comprehensive, person-centered care. Aligning workforce training, interdisciplinary care models, and behavioral health expertise could help to meet this challenge and improve the quality of life for some of the most complex and vulnerable patients receiving LTC.

## Supplementary Material

Supplemental 1

Supplemental 2

**Supplementary material.** The supplementary material for this article can be found at https://doi.org/10.1017/S1478951525100977.

## Figures and Tables

**Table 1.** Respondent characteristics

Variable	Category	*N* (%)
Age group	<30	69 (11.6%)
	31–40	142 (23.8%)
	41–50	143 (24.0%)
	51–60	151 (25.3%)
	61+	65 (10.9%)
	Prefer not to answer	25 (4.2%)
	Missing	2 (0.3%)
Racial/Ethnic group	White	150 (25.1%)
	Asian-American/Pacific Islander	66 (11.1%)
	Black	220 (36.9%)
	Hispanic/Latino	85 (14.2%)
	Other/Missing	19 (3.2%)
	Missing	57 (9.5%)
Gender	Man	108 (18.1%)
	Woman	475 (79.6%)
	Non-Binary/Unknown	13 (2.2%)
	Missing	1 (0.2%)
Discipline	RN	131 (21.9%)
	CNA/LPN	198 (33.2%)
	MD/NP/PA	34 (5.7%)
	Other	125 (20.9%)
	PT/OT/SLP	69 (11.6%)
	SW/Case manager	38 (6.4%)
	Missing	2 (0.3%)
Years in practice	<1	34 (5.7%)
	1–5	141 (23.6%)
	5–15	196 (32.8%)
	15+	217 (36.3%)
	Missing	9 (1.5%)
Palliative care experience	Professional	305 (51.1%)
	Personal	233 (39.0%)
Care setting	LTC	225 (37.7%)
	Multi-setting	204 (34.2%)
	Other	30 (5.0%)
	PACE	60 (10.1%)
	Subacute rehab	53 (8.9%)
	Missing	25 (4.2%)

Abbreviations: CNA: certified nursing assistant, LPN: licensed practical nurse, MD: Doctor of Medicine, NP: nurse practitioner, PA: physician assistant/physician association, PT: physical therapist, OT: occupational therapist, SLP: speech language pathologist, RN: registered nurse.

**Table 2.** Frequencies and percentages of respondents endorsing psychiatric symptom management questions

Statement	Frequency (%)
At least half of my patients would benefit from additional services in the domain of psychiatric symptom management	361 (60.5%)
I provide psychiatric symptom management weekly or more frequently in my clinical practice	294 (49.2%)
I feel comfortable or very comfortable providing psychiatric symptom management	291 (48.7%)
Psychiatric symptom management in top 3 ways a palliative care specialist could help patients	241 (40.4%)
Want to strengthen and expand psychiatric symptom management skills	266 (44.6%)
