# Supplementary material for: Behavioral health as a palliative care priority in long-term services and supports: A cross-sectional study of staff
Source: Palliat Support Care. 2025 Nov 4;23:e199. doi: 10.1017/S1478951525100977 (PMC12743431; doi:10.1017/S1478951525100977)
Supplement: Nowels et al. supplementary material 1 — Nowels et al. supplementary material [file S1478951525100977sup001.docx]

The ArchCare Palliative Care Survey

Thank you for taking the time to complete the Palliative Care Survey. Your responses will allow us to understand the Palliative Care needs at your facility and will help us shape the upcoming Palliative Care Education Program.

What is your age? < 21

21-30


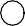

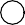

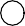

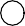

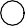

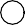

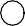


31-40

41-50

51-60

>61

Prefer not to answer

What is your gender? Male

Female Non-Binary


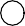

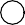

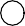

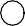


Prefer not to answer

Which of the following best describe you? Select all
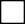
 Black or African American that apply.
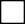
 Asian or Pacific Islander


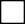
 Native American or Alaskan Native
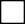
 Hispanic or Latino/a/x


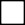
 White or Caucasian


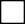
 A race or ethnicity not listed here
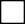
 Prefer not to answer

At what ArchCare site do you primarily work? (Check
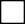
 Mary Manning Walsh Nursing Home

all that apply if appropriate)
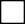
 Terence Cardinal Cooke Nursing Home
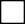
 Carmel Richmond Nursing Home


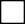
 Eger Nursing Home


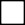
 St. Vincent De Paul Nursing Home
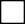
 Providence Rest Nursing Home


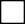
 Ferncliff Nursing Home
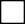
 Archcare at Home


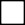
 ArchCare Community Life


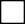
 ArchCare Senior Living--Harlem
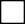
 ArchCare Senior Living--Cabrini
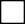
 Archcare Senior Living--Carmel


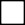
 ArchCare Senior Living--St. Vincent De Paul

In what setting(s) do you care for patients? Select
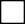
 Long-term care

all that apply.
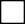
 Subacute rehab


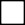
 PACE


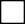
 Home
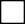
 Other

Please describe the other settings in which you provide care:

What is your discipline? Social worker


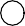

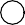

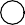

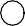

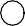

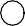

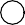

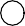

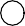

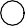

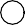

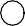


Registered Nurse Nurse Practitioner

Certified Nursing Assistant Licensed Practical Nurse Case Manager

Physician Assistant Physician

Physical Therapy Occupational Therapy Speech-Language Pathology Other

Please describe your discipline:

What is your specialty? Internal Medicine

Family Medicine Psychiatry


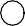

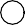

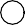

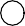

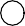

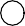

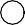

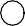

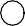

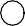

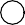


Physical Medicine and Rehabilitation Neurology

Surgery Ob-Gyn

Surgical Subspecialty Medical Subspecialty Geriatrics

Other

What is your specialty? Adult-Gerontology Nurse Practitioner (A-GNP) Psychiatric-Mental Health Nurse Practitioner (PMHNP)

Family Nurse Practitioner (FNP) Other


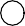

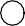

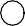

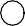


Please describe your specialty:

How many years of experience do you have working in < 1 your discipline? 1-5


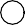

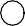

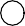

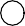


5-15

15+

How long have you been working at ArchCare? < 1 year 1-5 years

6-10 years


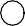

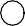

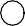

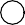

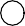


11-15 years

>15 years

In addition to your clinical work, do you also have
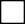
 Nurse Manager

any administrative roles?
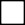
 Medical Director


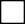
 Other


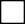
 None of the above

Please describe what other administrative role(s) you hold:

Are you currently in a formal clinical training Yes

program? (e.g., fellowship, clinical degree program)? No


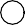

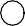


Please describe your training program:

What has been your prior exposure to palliative care?
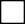
 Fellowship or subspecialty training

Select all that apply
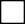
 Board-certification in hospice and palliative medicine or the equivalent for my discipline


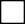
 Other certificate program


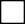
 Classes, lectures, or courses


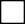
 Clinical rotation in palliative care


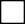
 Personal experience (for example, having a family member or friend who has received palliative care)


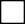
 Other


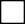
 I have not had exposure to palliative care

Please describe your exposure to palliative care:

Palliative Care Knowledge and Attitudes

Palliative care can improve the quality-of-life of my 1: Strongly 3: Neither agree

patients with serious illnesses disagree nor disagree 5: Strongly agree

*(Place a mark on the scale above)*

| I worry that consulting palliative care will make my | 1: Strongly | 3: Neither agree |  |
| --- | --- | --- | --- |
| patients lose hope | disagree | nor disagree | 5: Strongly agree |

*(Place a mark on the scale above)*

| I would like to increase access to palliative care | 1: Strongly | 3: Neither agree |  |
| --- | --- | --- | --- |
| services for my patients | disagree | nor disagree | 5: Strongly agree |

*(Place a mark on the scale above)*

Palliative care is another name for hospice care
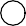
 Yes
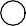
 No
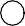
 Unsure

Patients can receive palliative care while they receive curative or life-prolonging treatment for their illness (e.g., chemotherapy for a cancer diagnosis)


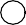
 Yes
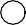
 No
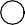
 Unsure

Practices and Needs

Do you check for the presence of advance care planning documents (e.g., health care proxy forms, medical orders for life sustaining treatment forms) when you begin to take care of a patient ?

Never Rarely Sometimes Often Always


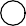

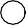

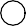

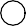

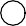

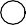


I'm not familiar with advance care planning

Do you ever refer to or consult palliative care? Yes


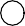

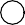


No, we do not have palliative care available No, I don't find their services helpful

No, I am able to provide this care myself No, I am not in a role where I would make referrals or consultations

Do you ever refer to or suggest hospice care? Yes

No, we do not have hospice services available No, I don't find hospice services helpful

No, I am able to provide this care myself No, I am not in a role where I would make referrals or consultations

**What percentage of patients in your care do you think would benefit from additional services**

**in the domains described below?**

Pain management

Non-pain symptom managment (e.g., nausea, constipation, shortness of breath)

< 10% 11-25% 26-50% 50-74% 75-90% 90-100% I do not

know/not applicable

Psychiatric symptom management (e.g., depression, anxiety, challenging behavior, confusion)

Caregiver and family support

Advance care planning and goals of care discussions

Spiritual care

Hospice referral and end-of-life care

Prognostication

Managing ethical conflicts

**Note: If you are in a role where any of these components of care are not applicable to you,**

**please select never.**

**How often do you provide the following components of care in your clinical practice?**

Pain management

Non-pain symptom management (e.g., nausea, constipation, shortness of breath)

Daily Multiple times a week

Weekly Monthly Less than once a month

Never

Psychiatric symptom management (e.g., depression, anxiety, challenging behavior, confusion)

Caregiver and family support

Advance care planning and goals of care discussions

Spiritual care

Hospice referral and end-of-life care

Prognostication

Managing ethical conflicts

**How comfortable do you feel providing the following components of care?**

Pain management

Non-pain symptom management (e.g., nausea, constipation, shortness of breath)

Very Uncomfortabl e

Uncomfortabl e

Neither Comfortable nor Uncomfortabl

Comfortable Very Comfortable

Not applicable

Psychiatric symptom management (e.g., depression, anxiety, challenging behavior, confusion)

e

Caregiver and family support

Advance care planning and goals of care discussions

Spiritual care

Hospice referral and end-of-life care

Prognostication

Managing ethical conflicts

**How helpful would the following be in caring for your patients with serious illnesses like**

**cancer or heart failure?**

A team of palliative care specialists who can see (consult on) selected patients

Not at all helpful

Slightly helpful

Moderately helpful

Helpful Very helpful I don't know

A palliative care clinician as part of my team or on my unit with

me

Training to help all clinicians to provide palliative care

Palliative care on-demand coaching (e.g., a number I could call to discuss a case and get tips)

Overall, what percentage of your patients do you believe would benefit from seeing a palliative care clinician (a physician, nurse, social worker, and/or chaplain working as part of a specialized palliative

care team)? 0 50 100

*(Place a mark on the scale above)*

What are the three most common ways that a palliative care specialist could help your patients?

Pain management

Non-pain symptom management (e.g., nausea, constipation, shortness of breath) Psychiatric symptom management (e.g., depression, anxiety, delirium)

Caregiver and family support

Advance care planning and goals of care discussions Spiritual care

Hospice referral and end-of-life care Prognostication

Managing ethical conflicts

Thinking about your current knowledge and skills, which of the following would be most useful to strengthen and expand them (select all you feel would be helpful):

Pain management

Non-pain symptom management (e.g., nausea, constipation, shortness of breath) Psychiatric symptom management (e.g., depression, anxiety, delirium)

Caregiver and family support

Advance care planning and goals of care discussions Spiritual care

Hospice referral and end-of-life care Prognostication

Managing ethical conflicts

Thinking about how best you learn, which of the following learning formats are most effective for you?

Dedicated lectures Computer modules

Having an expert available in my clinical setting to discuss cases Reading materials

Conferences or workshops Other

Please describe what other learning methods you find helpful:

Please provide your name. Your name will not be linked

to your responses and will be used only to ensure you receive credit (including care coins) for completing

the survey.
