# Supplementary material for: Behavioral health as a palliative care priority in long-term services and supports: A cross-sectional study of staff
Source: Palliat Support Care. 2025 Nov 4;23:e199. doi: 10.1017/S1478951525100977 (PMC12743431; doi:10.1017/S1478951525100977)
Supplement: Nowels et al. supplementary material 2 — Nowels et al. supplementary material [file S1478951525100977sup002.pdf]

The ArchCare Palliative Care Survey

Thank you for taking the time to complete the Palliative Care Survey. Your responses will allow us to understand the Palliative Care needs at your facility and will help us shape the upcoming Palliative Care Education Program.

What is your age?

- ○ < 21
- ○ 21-30
- ○ 31-40
- ○ 41-50
- ○ 51-60
- ○ >61
- ○ Prefer not to answer

What is your gender?

- ○ Male
- ○ Female
- ○ Non-Binary
- ○ Prefer not to answer

Which of the following best describe you? Select all that apply.

- ☐ Black or African American
- ☐ Asian or Pacific Islander
- ☐ Native American or Alaskan Native
- ☐ Hispanic or Latino/a/x
- ☐ White or Caucasian
- ☐ A race or ethnicity not listed here
- ☐ Prefer not to answer

At what ArchCare site do you primarily work? (Check all that apply if appropriate)

- ☐ Mary Manning Walsh Nursing Home
- ☐ Terence Cardinal Cooke Nursing Home
- ☐ Carmel Richmond Nursing Home
- ☐ Eger Nursing Home
- ☐ St. Vincent De Paul Nursing Home
- ☐ Providence Rest Nursing Home
- ☐ Ferncliff Nursing Home
- ☐ Archcare at Home
- ☐ ArchCare Community Life
- ☐ ArchCare Senior Living--Harlem
- ☐ ArchCare Senior Living--Cabrini
- ☐ Archcare Senior Living--Carmel
- ☐ ArchCare Senior Living--St. Vincent De Paul

In what setting(s) do you care for patients? Select all that apply.

- ☐ Long-term care
- ☐ Subacute rehab
- ☐ PACE
- ☐ Home
- ☐ Other

Please describe the other settings in which you provide care:

____

What is your discipline?	○ Social worker<br>○ Registered Nurse<br>○ Nurse Practitioner<br>○ Certified Nursing Assistant<br>○ Licensed Practical Nurse<br>○ Case Manager<br>○ Physician Assistant<br>○ Physician<br>○ Physical Therapy<br>○ Occupational Therapy<br>○ Speech-Language Pathology<br>○ Other
Please describe your discipline:	__________________________________
What is your specialty?	○ Internal Medicine<br>○ Family Medicine<br>○ Psychiatry<br>○ Physical Medicine and Rehabilitation<br>○ Neurology<br>○ Surgery<br>○ Ob-Gyn<br>○ Surgical Subspecialty<br>○ Medical Subspecialty<br>○ Geriatrics<br>○ Other
What is your specialty?	○ Adult-Gerontology Nurse Practitioner (A-GNP)<br>○ Psychiatric-Mental Health Nurse Practitioner (PMHNP)<br>○ Family Nurse Practitioner (FNP)<br>○ Other
Please describe your specialty:	__________________________________
How many years of experience do you have working in your discipline?	○ < 1<br>○ 1-5<br>○ 5-15<br>○ 15+
How long have you been working at ArchCare?	○ < 1 year<br>○ 1-5 years<br>○ 6-10 years<br>○ 11-15 years<br>○ >15 years
In addition to your clinical work, do you also have any administrative roles?	☐ Nurse Manager<br>☐ Medical Director<br>☐ Other<br>☐ None of the above
Please describe what other administrative role(s) you hold:	__________________________________
Are you currently in a formal clinical training program? (e.g., fellowship, clinical degree program)?	○ Yes<br>○ No

Please describe your training program: __________________________________

What has been your prior exposure to palliative care?
Select all that apply

- ☐ Fellowship or subspecialty training
- ☐ Board-certification in hospice and palliative medicine or the equivalent for my discipline
- ☐ Other certificate program
- ☐ Classes, lectures, or courses
- ☐ Clinical rotation in palliative care
- ☐ Personal experience (for example, having a family member or friend who has received palliative care)
- ☐ Other
- ☐ I have not had exposure to palliative care

Please describe your exposure to palliative care: __________________________________

Palliative Care Knowledge and Attitudes

Palliative care can improve the quality-of-life of my patients with serious illnesses

1: Strongly disagree — 3: Neither agree nor disagree — 5: Strongly agree

(Place a mark on the scale above)

I worry that consulting palliative care will make my patients lose hope

1: Strongly disagree — 3: Neither agree nor disagree — 5: Strongly agree

(Place a mark on the scale above)

I would like to increase access to palliative care services for my patients

1: Strongly disagree — 3: Neither agree nor disagree — 5: Strongly agree

(Place a mark on the scale above)

Palliative care is another name for hospice care

◯ Yes ◯ No ◯ Unsure

Patients can receive palliative care while they receive curative or life-prolonging treatment for their illness (e.g., chemotherapy for a cancer diagnosis)

◯ Yes ◯ No ◯ Unsure

Practices and Needs

Do you check for the presence of advance care planning documents (e.g., health care proxy forms, medical orders for life sustaining treatment forms) when you begin to take care of a patient ?

- ◯ Never
- ◯ Rarely
- ◯ Sometimes
- ◯ Often
- ◯ Always
- ◯ I'm not familiar with advance care planning

Do you ever refer to or consult palliative care?

- ◯ Yes
- ◯ No, we do not have palliative care available
- ◯ No, I don't find their services helpful
- ◯ No, I am able to provide this care myself
- ◯ No, I am not in a role where I would make referrals or consultations

Do you ever refer to or suggest hospice care?

- ◯ Yes
- ◯ No, we do not have hospice services available
- ◯ No, I don't find hospice services helpful
- ◯ No, I am able to provide this care myself
- ◯ No, I am not in a role where I would make referrals or consultations

What percentage of patients in your care do you think would benefit from additional services in the domains described below?

	< 10%	11-25%	26-50%	50-74%	75-90%	90-100%	I do not know/not applicable
Pain management	◯	◯	◯	◯	◯	◯	◯
Non-pain symptom managment (e.g., nausea, constipation, shortness of breath)	◯	◯	◯	◯	◯	◯	◯
Psychiatric symptom management (e.g., depression, anxiety, challenging behavior, confusion)	◯	◯	◯	◯	◯	◯	◯
Caregiver and family support	◯	◯	◯	◯	◯	◯	◯
Advance care planning and goals of care discussions	◯	◯	◯	◯	◯	◯	◯
Spiritual care	◯	◯	◯	◯	◯	◯	◯
Hospice referral and end-of-life care	◯	◯	◯	◯	◯	◯	◯
Prognostication	◯	◯	◯	◯	◯	◯	◯
Managing ethical conflicts	◯	◯	◯	◯	◯	◯	◯

How often do you provide the following components of care in your clinical practice?

Note: If you are in a role where any of these components of care are not applicable to you, please select never.

	Daily	Multiple times a week	Weekly	Monthly	Less than once a month	Never
Pain management	◯	◯	◯	◯	◯	◯
Non-pain symptom management (e.g., nausea, constipation, shortness of breath)	◯	◯	◯	◯	◯	◯

Psychiatric symptom management (e.g., depression, anxiety, challenging behavior, confusion)	○	○	○	○	○	○
Caregiver and family support	○	○	○	○	○	○
Advance care planning and goals of care discussions	○	○	○	○	○	○
Spiritual care	○	○	○	○	○	○
Hospice referral and end-of-life care	○	○	○	○	○	○
Prognostication	○	○	○	○	○	○
Managing ethical conflicts	○	○	○	○	○	○

How comfortable do you feel providing the following components of care?

	Very Uncomfortable	Uncomfortable	Neither Comfortable nor Uncomfortable	Comfortable	Very Comfortable	Not applicable
Pain management	○	○	○	○	○	○
Non-pain symptom management (e.g., nausea, constipation, shortness of breath)	○	○	○	○	○	○
Psychiatric symptom management (e.g., depression, anxiety, challenging behavior, confusion)	○	○	○	○	○	○
Caregiver and family support	○	○	○	○	○	○
Advance care planning and goals of care discussions	○	○	○	○	○	○
Spiritual care	○	○	○	○	○	○
Hospice referral and end-of-life care	○	○	○	○	○	○
Prognostication	○	○	○	○	○	○
Managing ethical conflicts	○	○	○	○	○	○

How helpful would the following be in caring for your patients with serious illnesses like cancer or heart failure?

	Not at all helpful	Slightly helpful	Moderately helpful	Helpful	Very helpful	I don't know
A team of palliative care specialists who can see (consult on) selected patients	○	○	○	○	○	○
A palliative care clinician as part of my team or on my unit with me	○	○	○	○	○	○

Training to help all clinicians to provide palliative care	○	○	○	○	○	○
Palliative care on-demand coaching (e.g., a number I could call to discuss a case and get tips)	○	○	○	○	○	○

Overall, what percentage of your patients do you believe would benefit from seeing a palliative care clinician (a physician, nurse, social worker, and/or chaplain working as part of a specialized palliative care team)?

0 50 100

(Place a mark on the scale above)

What are the three most common ways that a palliative care specialist could help your patients?

☐ Pain management
☐ Non-pain symptom management (e.g., nausea, constipation, shortness of breath)
☐ Psychiatric symptom management (e.g., depression, anxiety, delirium)
☐ Caregiver and family support
☐ Advance care planning and goals of care discussions
☐ Spiritual care
☐ Hospice referral and end-of-life care
☐ Prognostication
☐ Managing ethical conflicts

Thinking about your current knowledge and skills, which of the following would be most useful to strengthen and expand them (select all you feel would be helpful):

☐ Pain management
☐ Non-pain symptom management (e.g., nausea, constipation, shortness of breath)
☐ Psychiatric symptom management (e.g., depression, anxiety, delirium)
☐ Caregiver and family support
☐ Advance care planning and goals of care discussions
☐ Spiritual care
☐ Hospice referral and end-of-life care
☐ Prognostication
☐ Managing ethical conflicts

Thinking about how best you learn, which of the following learning formats are most effective for you?

☐ Dedicated lectures
☐ Computer modules
☐ Having an expert available in my clinical setting to discuss cases
☐ Reading materials
☐ Conferences or workshops
☐ Other

Please describe what other learning methods you find helpful:

____

Please provide your name. Your name will not be linked to your responses and will be used only to ensure you receive credit (including care coins) for completing the survey.

____
